# A safety framework for flow decomposition problems via integer linear programming

**DOI:** 10.1093/bioinformatics/btad640

**Published:** 2023-10-20

**Authors:** Fernando H C Dias, Manuel Cáceres, Lucia Williams, Brendan Mumey, Alexandru I Tomescu

**Affiliations:** Department of Computer Science, University of Helsinki, Helsinki 00560, Finland; Department of Computer Science, University of Helsinki, Helsinki 00560, Finland; School of Computing, Montana State University, Bozeman, MT 59717, United States; School of Computing, Montana State University, Bozeman, MT 59717, United States; Department of Computer Science, University of Helsinki, Helsinki 00560, Finland

## Abstract

**Motivation:**

Many important problems in Bioinformatics (e.g. assembly or multiassembly) admit multiple solutions, while the final objective is to report only one. A common approach to deal with this uncertainty is finding “safe” partial solutions (e.g. contigs) which are common to all solutions. Previous research on safety has focused on polynomially time solvable problems, whereas many successful and natural models are NP-hard to solve, leaving a lack of “safety tools” for such problems. We propose the first method for computing all safe solutions for an NP-hard problem, “minimum flow decomposition” (MFD). We obtain our results by developing a “safety test” for paths based on a general integer linear programming (ILP) formulation. Moreover, we provide implementations with practical optimizations aimed to reduce the total ILP time, the most efficient of these being based on a recursive group-testing procedure.

**Results:**

Experimental results on transcriptome datasets show that all safe paths for MFDs correctly recover up to 90% of the full RNA transcripts, which is at least 25% more than previously known safe paths. Moreover, despite the NP-hardness of the problem, we can report all safe paths for 99.8% of the over 27 000 non-trivial graphs of this dataset in only 1.5 h. Our results suggest that, on perfect data, there is less ambiguity than thought in the notoriously hard RNA assembly problem.

**Availability and implementation:**

https://github.com/algbio/mfd-safety.

## 1 Introduction

In real-world scenarios where an unknown object needs to be discovered from the input data, we would like to formulate a computational problem loosely enough so that the unknown object is indeed a solution to the problem, but also tightly enough so that the problem does not admit many other solutions. However, this goal is difficult in practice, and indeed, various commonly used problem formulations in Bioinformatics still admit many solutions. While a naive approach is to just exhaustively enumerate all these solutions, a more practical approach is to report only those subsolutions (or partial solutions) that are common to “all” solutions to the problem.

In the graph theory community such subsolutions have been called “persistent” (Hammer *et al.* 1982, Costa 1994), and in the Bioinformatics community “reliable” (Vingron and Argos 1990), or more recently, “safe” (Tomescu and Medvedev 2017). The study of safe subsolutions started in Bioinformatics in the 1990s (Vingron and Argos 1990, Chao *et al.* 1993, Naor and Brutlag 1994) with those amino-acid pairs that are common to all optimal and suboptimal alignments of two protein sequences.

In the genome assembly community, the notion of “contig,” namely a string that is guaranteed to appear in any possible assembly of the reads, is at the core of most genome assemblers. This approach originated in 1995 with the notion of unitigs (Kececioglu and Myers 1995) (nonbranching paths in an assembly graph), which were progressively (Pevzner *et al.* 2001, Bonnici *et al.* 2021) generalized to paths made up of a prefix of nodes with in-degree one followed by nodes without-degree one (Medvedev *et al.* 2007, Jackson 2009, Kingsford *et al.* 2010) (also called extended unitigs or Y-to-V contigs).

Later, Tomescu and Medvedev (2017) formalized all such types of contigs as those “safe” strings that appear in all solutions to a genome assembly problem formulation, expressed as a certain type of walk in a graph. Cairo *et al.* (2019, 2023) proposed more efficient and unifying safety algorithms for several types of graph walks. Rahman and Medvedev (2022) recently studied the safety of contigs produced by state-of-the-art genome assemblers on real data.

Analogous studies were recently made also for multiassembly problems, where several related genomic sequences need to be assembled from a sample of mixed reads. Cáceres *et al.* (2022a) studied safe paths that appear in all constrained path covers of a directed acyclic graph (DAG). Zheng, Ma, and Kingsford studied the more practical setting of a network flow in a DAG by finding those paths that appear in any flow decomposition (FD) of the given network flow, under a probabilistic framework (Ma *et al.* 2021) or a combinatorial framework (Zheng *et al.* 2022). (The problem AND-Quant from Zheng *et al.* (2022) actually handles a more general version of this problem.) Khan *et al.* (2022b) presented a simple characterization of safe paths appearing in any FD of a given acyclic network flow, leading to a more efficient algorithm than the one of Zheng *et al.* (2022) and further optimized by Khan and Tomescu (2022).

### 1.2 Motivation

Despite the significant progress in obtaining safe algorithms for a range of different applications, current safe algorithms are limited to problems where computing a solution itself is achievable in polynomial time. However, many natural problems are NP-hard, and safe algorithms for such problems are fully missing. Apart from the theoretical interest, usually such NP-hard problems correspond to restrictions of easier (polynomially computable) problems, and thus by definition, also have longer safe subsolutions.

As such, current safety algorithms miss data that could be reported as correct, just because they do not constrain the solution space enough. A major reason for this lack of progress is that if a problem is NP-hard, then its safety version is likely to be hard too. This phenomenon can be found both in classically studied NP-hard problems—for example, computing nodes present in all maximum independent sets of an undirected graph is NP-hard (Hammer *et al.* 1982)—as well as in NP-hard problems studied for their application to Bioinformatics, as we discuss further in the Supplementary Material.

We introduce our results by focusing on the “FD problem.” This is a classical model at the core of multiassembly software for RNA transcripts (Li *et al.* 2011a, b, Tomescu *et al.* 2013, Bernard *et al.* 2014) and viral quasi-species genomes (Chen *et al.* 2018, Baaijens *et al.* 2019, 2020, Posada-Céspedes *et al.* 2021), and also a standard problem with applications in other fields, such as networking (Hartman *et al.* 2012, Hong *et al.* 2013, Cohen *et al.* 2014, Mumey *et al.* 2015) or transportation (Ohst 2015, Olsen *et al.* 2022). In its most basic optimization form, “minimum FD” (MFD), we are given a flow in a graph, and we need to decompose it into a minimum number of paths with associated weights, such that the superposition of these weighted paths gives the original flow. This is an NP-hard problem, even when restricted to DAGs (Vatinlen *et al.* 2008, Hartman *et al.* 2012). Various approaches have been proposed to tackle the problem, including fixed-parameter tractable algorithms (Kloster *et al.* 2018), approximation algorithms (Mumey *et al.* 2015, Cáceres *et al.* 2022b), and integer linear programming (ILP) formulations (Sashittal *et al.* 2021, Dias *et al.* 2022b). In Bioinformatics applications, reads, or contigs originating from a mixed sample of genomic sequences, with different abundances, are aligned to a reference. A graph model, such as a splice graph or a variation graph, is built from these alignments. Read abundances assigned to the nodes and edges of this graph then correspond to a flow in case of perfect data. If this is not the case, then the abundance values can either be minimally corrected to become a flow or one can consider variations of the problem where, e.g. the superposition of the weighted paths is closest (or within a certain range) to the edge abundances (Tomescu *et al.* 2013, Bernard *et al.* 2014).

Current safety algorithms for FDs such as (Khan *et al.* 2022a, b, Khan and Tomescu 2022, Zheng *et al.* 2022) compute paths appearing in *all* possible FDs (of any size), even though decompositions of minimum size are assumed to better model the RNA assembly problem (Shao and Kingsford 2017b, Kloster *et al.* 2018, Williams *et al.* 2021). Even dropping the minimality constraint, but adding other simple constraints easily renders the problem NP-hard (see e.g. Williams *et al.* 2022), motivating further study of practical safe algorithms for NP-hard problems.

### 1.3 Contributions

ILP is a general and flexible method that has been successfully applied to solve NP-hard problems, including in Bioinformatics. In this article, we consider graph problems whose solution consists of a set of “paths” (i.e. not repeating nodes) that can be formulated in ILP. We introduce a technique that, given an ILP formulation of such a graph problem, can enhance it with additional variables and constraints in order to test the safety of a given set of paths. An obvious first application of this safety test is to use it with a single path in a straightforward avoid-and-test approach, using a standard two-pointer technique that has been used previously to find safe paths for FD. However, we find that a top-down recursive approach that uses the group-testing capability halves the number of computationally-intensive ILP calls, resulting in a 3× speedup over the straightforward approach.

Additionally, we prove that computing all the safe paths for MFDs is an intractable problem, confirming the above intuitive claim that if a problem is hard, then also its safety version is hard. We give this proof in the Supplementary Material by showing that the NP-hardness reduction for MFD by Hartman *et al.* (2012) can be modified into a Turing reduction from the UNIQUE 3SAT problem.

On the dataset, Shao and Kingsford (2017a) containing splice graphs from human, zebrafish, and mouse transcriptomes, safe paths for MFDs (*SafeMFD*) correctly recover up to 90% of the full RNA transcripts while maintaining a 99% precision, outperforming, by a wide margin (25% increase), state-of-the-art safety approaches, such as extended unitigs (Medvedev *et al.* 2007, Jackson 2009, Kingsford *et al.* 2010), safe paths for constrained path covers of the edges (Cáceres *et al.* 2022a), and safe paths for all FDs (Khan and Tomescu 2022, Khan *et al.* 2022a, b, Zheng *et al.* 2022). On a harder dataset by Khan *et al.* (2022a), *SafeMFD* also dominates in a significant proportion of splice graphs (built from t≤15 RNA transcripts), recovering more than 93% of the full transcripts while maintaining a 98% precision. For larger *t*, precision drastically drops (91% precision in the entire dataset), suggesting that in more complex splice graphs smaller solutions are introduced as an artifact of the combinatorial nature of the splice graph, and the minimality condition (Shao and Kingsford 2017b, Kloster *et al.* 2018, Williams *et al.* 2021) is thus incorrect in this domain. On a third dataset, created from real human RNA reads (Dias *et al.* 2022b), again we found that the precision of *SafeMFD* drops as the number of transcripts grows, again refuting the assumption of minimum decomposition (see Supplementary Fig. S5). For each dataset, we also tested the effect of providing additional subpath constraints (subpaths that are known to be part of the solution paths, see Williams *et al.* 2021); such constraints improved the solutions found by 1–3 percentage points.

## 2 Methods

### 2.1 Preliminaries

#### 2.1.1 ILP models

In this article, we use ILP models as blackboxes, with as few assumptions as possible to further underline the generality of our approach. Let M(V,C) be an ILP model consisting of a set V of variables and a set C of constraints on these variables, built from an input graph G=(V,E). We make only two assumptions on M. First, that a solution to this model consists of a given number k≥1 of paths $P_{1},\ldots ,P_{k}$ in *G* (in this article, paths do not repeat vertices). Second, we assume that the *k* paths are modeled via binary edge variables $x_{\mathrm{uvi}}$, for all (u,v)∈E and for all i∈{1,…,k}. More specifically, for all i∈{1,…,k}, we require that the edges (u,v)∈E for which the corresponding variable $x_{\mathrm{uvi}}$ equals 1 induce a path in *G*. For example, if *G* is a DAG, it is a standard fact (see e.g. Taccari 2016) that a path from a given s∈V to a given t∈V (an *s-t path*) can be expressed with the following constraints:


(1) $$\sum_{(u,v)\in E}x_{\mathrm{uvi}}-\sum_{(v,u)\in E}x_{\mathrm{vui}}=\{\begin{array}{cc} 0, & \text{if}\;v\in V\setminus \{s,t\},\\ 1, & \text{if}\;v=t,\\ -1, & \text{if}\;v=s. \end{array}$$

If *G* is not a DAG, there are other types of constraints that can be added to the $x_{\mathrm{uvi}}$ variables to ensure that they induce a path; see, e.g. the many formulations in Taccari (2016). We will assume that such constraints are part of the set C of constraints of M(V,C), but their exact formulation is immaterial for our approach. In fact, one could even add additional constraints to C to further restrict the solution space. For example, some ILP models from Dias *et al.* (2022b) and Sashittal *et al.* (2021) handle the case when the input also contains a set of paths (*subpath constraints*) that must appear in at least one of the *k* solution paths; we will explain these constraints later.

#### 2.1.2 Flow decomposition

**Figure 1.**
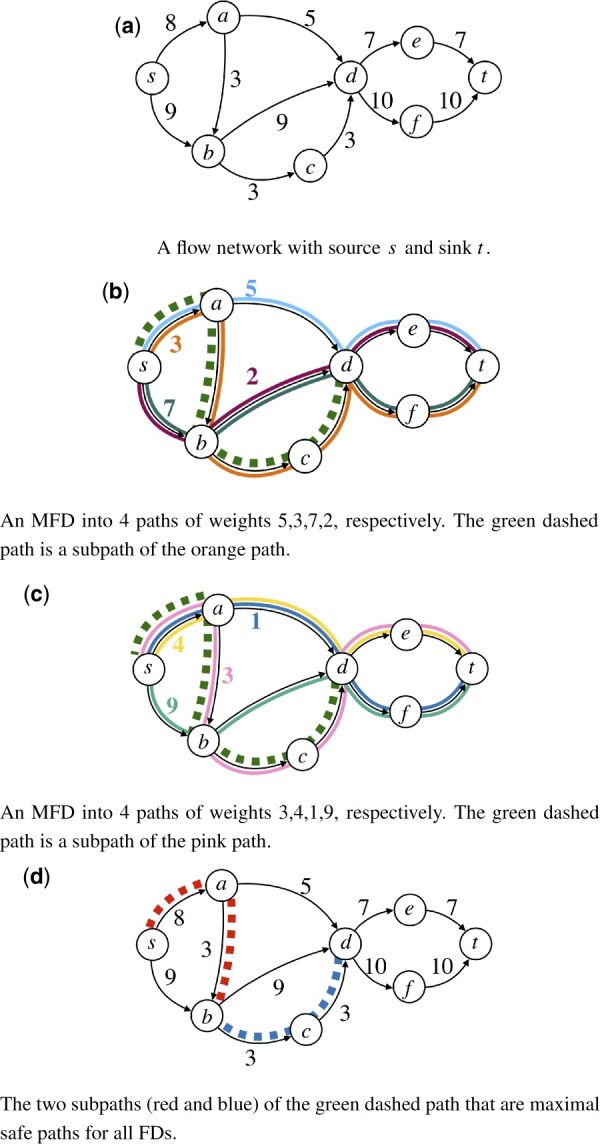
Flow decompositions and safe paths. The flow network in (a) admits different MFDs, in (b) and in (c). The path (s,a,b,c,d) (dashed green) is a maximal safe path for MFDs, i.e. it is a subpath of some path of all MFDs and it cannot be extended without losing this property. However, the path (s,a,b,c,d) is not safe for all FDs. Indeed, its two subpaths (s,a,b) (dashed red in (d)) and (b,c,d) (dashed blue in (d)) are maximal safe paths for all FDs. To see this, note that the excess flow of (s,a,b) is 3, while the excess flow of (s,a,b,c) (and of (s,a,b,c,d)) is −6

In the FD problem we are given a flow network (V,E,f), where G=(V,E) is a (directed) graph with unique source s∈V and unique sink t∈V, and *f* assigns a positive integer “flow value” $f_{uv}$ to every edge (u,v)∈E. “Flow conservation” must hold for every node different from *s* and *t*, namely, the sum of the flow values entering the node must equal the sum of the flow values exiting the node. See Fig. 1(a) for an example. We say that k s-*t* paths $P_{1},\ldots ,P_{k}$, with associated positive integer weights $w_{1},\ldots ,w_{k}$, are a *FD* if their superposition equals the flow *f*. Formally, for every (u,v)∈E it must hold that


(2) $$\sum_{\overset{i\in \{1,\ldots ,k\}\;s.t.}{(u,v)\in P_{i}}}w_{i}=f_{uv}.$$

See Fig. 1(b) and (c) for two examples. The number *k* of paths is also called the “size” of the FD. In the “MFD problem,” we need to find a FD of minimum size. (In this article, we work only with integer flow values and weights for simplicity and since this is the most studied version of the problem, see e.g. Kloster *et al.* (2018). However, the problem can also be defined with fractional weights (Pertea *et al.* 2015), and in this case the two problems can have different minima on the same input (Vatinlen *et al.* 2008). This fractional case can also be modeled by ILP (Dias *et al.* 2022b), and all the results from our article also immediately carry over to this variant.) On DAGs, a FD into paths always exists (Ahuja *et al.* 1988), but in general graphs, cycles may be necessary to decompose the flow [see e.g. Dias *et al.* (2022a) for different possible formulations of the problem].

For concreteness, we now describe the ILP models from Dias *et al.* (2022b) for finding a FD into *k* weighted paths in a DAG. They consist of (i) modeling the *k* paths via the $x_{\mathrm{uvi}}$ variables [with constraints (1)], (ii) adding path-weight variables $w_{1},\ldots ,w_{k}$, and (iii) requiring that these weighted paths form a FD, via the following (nonlinear) constraint:


(3) $$\begin{array}{cc} \sum_{i\in \{1,\ldots ,k\}}x_{\mathrm{uvi}}w_{i}=f_{uv}, & \forall (u,v)\in E. \end{array}$$

This constraint can then be easily linearized by introducing additional variables and constraints; see e.g. Dias *et al.* (2022b) for these technical details. However, as mentioned above, the precise formulation of the ILP model M for a problem is immaterial for our method. Only the two assumptions on M made above matter for obtaining our results.

#### 2.1.3 Subpath constraints

Additionally, the FD model can be further constrained by requiring specific input paths to be part of the solution (Dias *et al.* 2022b). These constraints can be modeled from RNA-seq long reads without rendering the RNA assembly problem obsolete, as mentioned by Zhang *et al.* (2021), Amarasinghe *et al.* (2020), and Voshall and Moriyama (2018). More formally, given a set of paths $R=\{R_{1},\ldots ,R_{\ell }\}$ of *G* (not necessarily *s*-*t* paths), we require that every path in R is a subpath of some $P_{i}$ in the decomposition. To represent this behavior in the ILP formulation, we add the following constraints to C:


(4a) $$\begin{array}{cc} \sum_{(u,v)\in R_{j}}x_{\mathrm{uvi}}\geq |R_{j}|\cdot r_{ij}, & \forall i\in \{1,\ldots ,k\},\forall j\in \{1,\ldots ,\ell \}, \end{array}$$

(4b) $$\sum_{i\in \{1,\ldots ,k\}}r_{ij}\geq 1,\forall j\in \{1,\ldots ,\ell \}.$$

More specifically, for each $R_{j}\in R$, we introduce an additional binary variable $r_{ij}$ denoting the presence of the subpath $R_{j}$ in the *i*th decomposition path. Constraints (4b) ensure that at least one decomposition path contains each subpath and constraints (4a) indicate whether subpath $R_{j}$ is present in the *i*th decomposition path.

#### 2.1.4 Safety

Given a problem on a graph *G* whose solutions consist of *k* paths in *G*, we say that a path *P* is safe if for any solution $P_{1},\ldots ,P_{k}$ to the problem, there exists some i∈{1,…,k} such that *P* is a subpath of $P_{i}$. If the problem is given as an ILP model M, we also say that *P* is safe for M. We say that *P* is a “maximal safe path,” if *P* is a safe path and there is no larger safe path containing *P* as subpath. Khan *et al.* (2022b) characterized safe paths for “all” FDs (not necessarily of minimum size) using the “excess flow” $f_{P}$ of a path *P*, defined as the flow on the first edge of *P* minus the flow on the edges out-going from the internal nodes of *P*, and different from the edges of *P* [see Fig. 1(d) for an example]. It holds that *P* is safe for all FDs if and only if $f_{P}>0$ (Khan *et al.* 2022b). The excess flow can be computed in time linear in the length of *P* (assuming we have precomputed the flow outgoing from every node), giving thus a linear-time verification of whether *P* is safe.

A basic property of safe solutions is that any sub-solution of them is also safe. Computing safe paths for MFDs can thus potentially lead to joining several safe paths for FDs, obtaining longer paths from the unknown sequences we are trying to assemble. See Fig. 1 for an example of a maximal safe path for MFDs and two maximal subpaths of it that are safe for FDs.

### 2.2 Finding maximal safe paths for MFD via ILP

We now present a method for finding all maximal safe paths for MFD via ILP. The basic idea is to define an inner “safety test” which can be repeatedly called as part of an outer algorithm over the entire instance to find all maximal safe paths. Because calls to the ILP solver are expensive, the guiding choice for our overall approach is to minimize the number of ILP calls. This inspires us to test the safety of a “group” of paths as the inner safety test, which we achieve by augmenting our ILP model so that it can give us information about the safety of the paths in the set. We use this to define a recursive algorithm to fully determine the safety status of each path in a group of paths. We can then structure the safety test in either a top-down manner (starting with long unsafe paths and shrinking them until they are safe) or a bottom-up manner (starting with short safe paths and lengthening them until they become unsafe).

#### 2.2.1 Safety test (inner algorithm)

Let M(V,C) be an ILP model as discussed in Section 2.1; namely, its *k* solution paths are modeled by binary variables $x_{\mathrm{uvi}}$ for each (u,v)∈E and each i∈{1,…,k}. We assume that M(V,C) is feasible (i.e. the problem admits at least one solution). We first show how to modify the ILP model so that, for a given set of paths, it can tell us one of the following: (1) a set of paths that are “not safe” (the remaining being of unknown status) or (2) that all paths are safe. The idea is to maximize the number of paths that can be simultaneously avoided from the given set of paths.

Let P be a set of paths. For each path P∈P, we create an auxiliary binary variable $\gamma_{P}$ that indicates:


(5) $$\gamma_{P}\equiv \{\begin{array}{cc} 1 & \text{if}\;P\;\text{was avoided in the solution},\\ 0 & \text{otherwise}. \end{array}$$

Since the model solutions are “paths” (i.e. not repeating nodes), we can encode whether *P* appears in the solution by whether all of the ℓ−1 edges of *P* appear simultaneously. Using this fact, we add a new set of constraints R(P) that include the $\gamma_{P}$ indicator variables for each path P∈P:


(6) $$\begin{array}{c} R(P):=\{x_{v_{1}v_{2}i}+x_{v_{2}v_{3}i}+\cdots +x_{v_{\ell -1}v_{\ell }i}\\ \;\leq \ell -1-\gamma_{P}:\forall i\in \{1,\ldots ,k\},\forall P\in P\}. \end{array}$$

**Figure 2.**
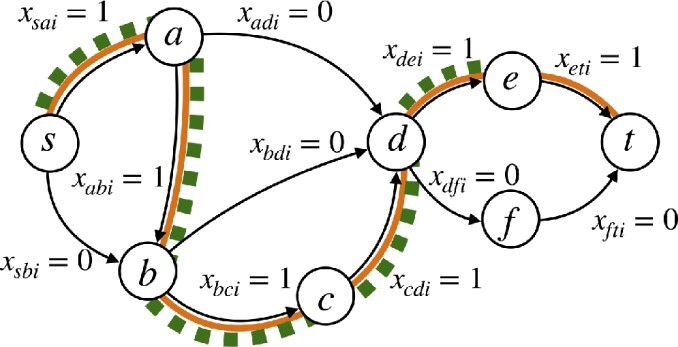
Illustration of modeling a solution path and a tested path via binary edge variables and safety verification constraints. The *i*th solution path $P_{i}$ is shown in orange and a tested path *P* is shown in dashed green. Constraint (6) includes $x_{\mathrm{sai}}+x_{\mathrm{abi}}+x_{\mathrm{bci}}+x_{\mathrm{cdi}}+x_{\mathrm{dei}}\leq 5-\gamma_{P}$. This simplifies to $\gamma_{P}\leq 0$, thus forcing $\gamma_{P}=0$, which indicates *P* was not avoided in the solution.

See Fig. 2 for an example. Next, as the objective function of the ILP model, we require that it should maximize the number of avoided paths from P, i.e. the sum of the $\gamma_{P}$ variables:


(7) $$\max\sum_{P\in P}\gamma_{P}.$$

All paths *P* such that $\gamma_{P}=1$ are “unsafe,” since they were avoided in some MFD. Conversely, if the objective value of Equation (7) was 0, then $\gamma_{P}=0$ for all paths in P, and it must be that all paths in P are safe (if not, at least one path could be avoided and increase the objective). We encapsulate this group-testing ILP in a function GroupTest(M,P) that returns a set N⊆P with the properties that: (1) if N=∅, then all paths in P are safe and (2) if N≠∅, then all paths in N are unsafe (and |N| is maximized).

We employ GroupTest(M,P) to construct a recursive procedure GetSafe(M,P) that determines all safe paths in P, as shown in Algorithm 1.

Algorithm 1. Testing a set of paths P for safety.

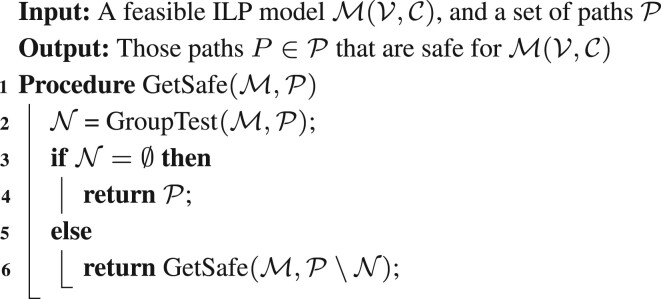

We note that in the special case that |P|=1, GetSafe(M,P) makes only a single call to the ILP via GroupTest(M,P) to determine whether not the given path is safe. With this safety test for a single path, we can easily adapt a standard two-pointer approach as the outer algorithm to find all maximal safe paths for MFD by starting with some MFD solution $P_{1},\ldots ,P_{k}$ of M(V,C). This same procedure was used in Khan *et al.* (2022a) to find all maximal safe paths for FD, using an excess flow check as the inner safety algorithm.

#### 2.2.2 Find all maximal safe paths (outer algorithm)

We give two algorithms for finding all maximal safe paths. Both algorithms use a similar approach, however the first uses a top-down approach starting from the original full solution paths and reports all safe paths (these again must be maximal safe), and then trims all the unsafe paths to find new maximal safe paths. The second is bottom-up in that it tries to extend known safe subpaths until they cannot be further extended (and at this point must be maximal safe). We present the first algorithm in detail and defer discussion of the second to the Supplementary Material.

We say a set of subpaths $T=\{P_{i}[l_{i},r_{i}]\}$ is a “trimming core” provided that for any unreported maximal safe path $P=P_{i}[l,r]$, there is a $P_{i}[l_{i},r_{i}]\in T$, where $l_{i}\leq l\leq r\leq r_{i}$.

Algorithm 2. An algorithm to compute all maximal safe paths that can be trimmed from a trimming core set T.

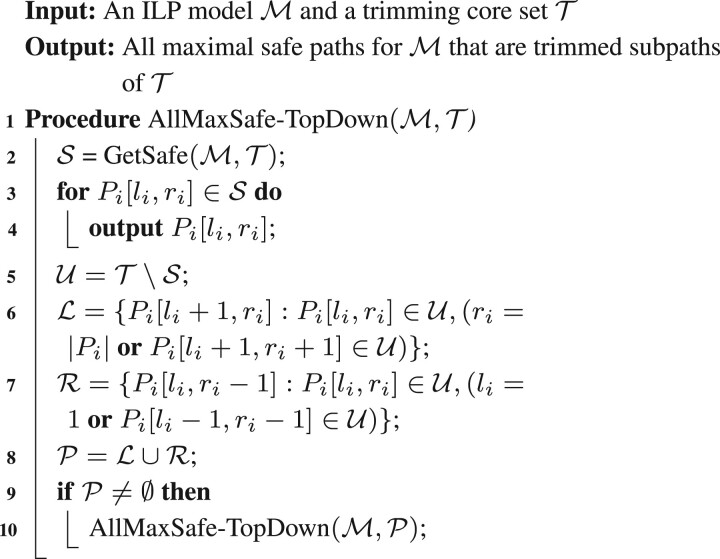

We will use the original *k* solution paths $\left\{P_{i}\right\}$ as our initial trimming core; the complete algorithm is given in Algorithm 2. See Supplementary Fig. S1 for an illustration of the algorithm’s initial steps. The algorithm first checks if any of the paths in T are safe; if so, these are reported as maximal safe. For those paths that were unsafe, it then considers trimming one vertex from the left and one vertex from the right to create new subpaths. Of these subpaths, some may be contained in a safe path in T; these subpaths can be ignored as they are not maximal safe. The algorithm recurses on those subpaths whose safety status cannot be determined (lines 6–10). In this way, the algorithm maintains the invariant that no paths in T are properly contained in a safe path; thus, paths reported in line 4 must be maximal safe.

## 3 Experiments

To test the performance of our methods, we computed safe paths using different safety approaches and reported the quality and running time performances as described below. Additional details on the experimental setup are given in the Supplementary Material.

### 3.1 Implementation details—SafeMFD

We implemented the previously described algorithms to compute all maximal safe paths for MFDs in Python. The implementation, *SafeMFD*, uses the package NetworkX (Hagberg *et al.* 2008) for graph processing and the package gurobipy (Gurobi Optimization, LLC 2021) to model and solve the ILPs and it is openly available (https://github.com/algbio/mfd-safety). Our fastest variant (see Supplementary Table S3 for a comparison of running times) implements Algorithm 2 using the group testing in Algorithm 1. We used this variant to compare against other safety approaches. All tested variants of *SafeMFD* implement the following two optimizations:

1. Before processing an input flow graph, we contract it using Y-to-V contraction (Tomescu and Medvedev 2017), which is known (Kloster *et al.* 2018) to maintain (M)FD solution paths. Moreover, since edges in the contracted graph correspond to extended unitigs (Medvedev *et al.* 2007, Jackson 2009, Kingsford *et al.* 2010), source-to-sink edges are further removed from the contracted graph and reported as safe. As such, our algorithms compute all maximal safe paths for funnels (Garlet Millani *et al.* 2020, Khan *et al.* 2022a) without using the ILP.
2. Before testing the safety of a path, we check if its “excess-flow” (Khan *et al.* 2022a) is positive. If this is the case, the path is removed from the corresponding test. Having positive excess flow implies safety for all FD and thus also safety for MFDs.

### 3.2 Safety approaches tested

We compare the following state-of-the-art safety approaches:

***EUnitigs*:** Maximal paths made up of a prefix of nodes with in-degree one followed by nodes with out-degree one; also called “extended unitigs” (Medvedev *et al.* 2007, Jackson 2009, Kingsford *et al.* 2010, Tomescu and Medvedev 2017). We use the C++ implementation provided by Khan *et al.* (2022a) (which computes only the extended unitigs contained in FD paths).

***SafeFlow*:** Maximal safe paths for all FDs (Khan *et al.* 2022a). We use the C++ implementation provided by the authors.

***SafeMFD*:** Maximal safe paths for all MFDs, as proposed in this work. Every flow graph processed is given a “time budget” of 2 min. If a flow graph consumes its time budget, the solution of “SafeFlow” is output instead.

***SafeEPC*:** Maximal safe paths for all constrained path covers of edges. Previous authors (Cáceres *et al.* 2022a, Khan *et al.* 2022a) have considered safe path covers of the nodes, but for a more fair comparison, we instead use path covers of edges. To this end, we transform the input graphs by splitting every edge by adding a node in the middle and run the C++ implementation provided by the authors of Cáceres *et al.* (2022a). Since FDs are path covers of edges, safe paths for all edge path covers are subpaths of safe paths for MFD. However, we restrict the path covers to those of minimum size and minimum size plus one, as recommended by the authors of Cáceres *et al.* (2022a) to obtain good coverage results while maintaining high precision.

All safety approaches require a post processing step for removing duplicates, prefixes, and suffixes. We use the C++ implementation provided by Khan *et al.* (2022a) for this purpose.

### 3.3 Datasets

We use three datasets of flow graphs inspired by RNA transcript assembly. The datasets were created by “perfectly” superposing a set of transcripts into splice graphs that are guaranteed to respect flow conservation. As such, the ground truth corresponds to a FD (not necessarily minimum), which is available for quality evaluation. To avoid a skewed picture of our results, we filtered out trivial instances with a unique FD (or funnels, see Garlet Millani *et al.* 2020, Khan *et al.* 2022a) from the two datasets. (The exact datasets used in our experiments can be found at https://zenodo.org/record/8275700.)

**Catfish:** Created by Shao and Kingsford (2017b), it includes 100 simulated human, mouse, and zebrafish transcriptomes using Flux-Simulator (Griebel *et al.* 2012) as well as 1000 experiments from the Sequence Read Archive simulating abundances using Salmon (Patro *et al.* 2015). We took one experiment per dataset, which corresponds to 27 696 nontrivial flow graphs.

**RefSim:** Created by Cáceres *et al.* (2022a) from the Ensembl (Yates *et al.* 2020) annotated transcripts of GRCh.104 *Homo sapiens* reference genome, and later augmented by Khan *et al.* (2022a) with simulated abundances using the RNASeqReadSimulator (Li 2014). This dataset has 10 323 nontrivial graphs.

**StringTie:** Created by Dias *et al.* (2022b) from real human RNA reads from SRR307903. From the reads, perfect splice graphs are produced by running HiSat2 (Kim *et al.* 2019) and StringTie (Kovaka *et al.* 2019). The resulting transcripts are used as ground truth. This dataset has 2187 nontrivial graphs.

We also evaluated the performance of “SafeMFD” when provided with subpath constraints. (These dataset were created to test the quality improvement of adding subpath constraints. Comparison of *SafeMFD* with other safe approaches ignores these constraints.) For each flow graph created from *t* transcripts, we take ⌊t/2⌋ transcripts and create one subpath constraint for each of those transcripts. The subpath constraints consist of the prefix of the corresponding transcript that includes three nontrivial junctions, as done by Dias *et al.* (2022b).

### 3.4 Quality metrics

We use the same quality metrics employed by previous multiassembly safety approaches (Cáceres *et al.* 2022a, Khan *et al.* 2022a). We provide a high-level description of them for completeness.

**Weighted precision of reported paths:** As opposed to normal precision, the weighted version considers the length of the reported subpaths. It is computed as the total length of the correctly reported subpaths divided by the total length of all reported subpaths. A reported subpath is considered “correct” if and only if it is a subpath of some path in the ground truth (exact alignment of exons/nodes).

**Maximum coverage of a ground truth path *P*:** The longest segment of *P* covered by some reported subpath (exact alignment of exons/nodes), divided by |P|.

We compute the “weighted precision of a graph” as the average weighted precision over all reported paths in the graph and the “maximum coverage of a graph” as the average maximum coverage over all ground truth paths in the graph.

***F*-Score of a graph:** Harmonic mean between weighted precision and maximum coverage of a graph, which assigns a global score to the corresponding approach on the graph.

These metrics are computed per flow graph and reported as an average. We report our metrics in terms of exons (nodes) for all datasets. When genomic loci of exons are available (datasets *StringTie* and *RefSim*) we also report the metrics in terms of genomic positions (see Supplementary Material).

## 4 Results and discussion

In the Catfish dataset, *EUnitigs* and *SafeFlow* ran in less than a second, while *SafeEPC* took approximately 30 s to compute. On the other hand, solving a harder problem, *SafeMFD* took approximately 1.5 h to compute in the rest of the dataset, timing out in only 54 graphs (we use a cutoff of 2 min), i.e. only 0.2% of the entire dataset. This equates to only 0.2 s on average per solved graph, underlying the scalability of our approach.

Table 1 shows that *SafeMFD*, on average, covers close to 90% of the ground truth paths, while maintaining a high precision (99%). This corresponds to an increase of approximately 25% in coverage against its closest competitor *SafeFlow*. *SafeMFD* also dominates in the combined metric of *F*-score, being the only safe approach with *F*-score over 90%. Supplementary Fig. S2 shows the metrics on graphs grouped by number *t* of ground truth paths, indicating the dominance in coverage and *F*-score of *SafeMFD* across all values of *t*, and indicating that the decrease in precision appears for large values of *t* (t≥12).

**Table 1.** Summary of quality metrics in terms of nodes/exons; *t* is the number of ground truth paths.

Dataset	Graphs	Algorithm	Max. Coverage	Wt. Precision	*F*-score
*Catfish*	All (100%)	EUnitigs	0.60	1.00	0.74
		SafeEPC	0.60	0.99	0.74
		SafeFlow	0.71	1.00	0.82
		SafeMFD	0.88	0.99	0.93
*StringTie*	All (100%)	EUnitigs	0.68	1.00	0.80
		SafeEPC	0.68	1.00	0.80
		SafeFlow	0.83	1.00	0.90
		SafeMFD	0.92	0.98	0.94
*RefSim*	t≤10 (68%)	EUnitigs	0.55	1.00	0.71
		SafeEPC	0.56	1.00	0.71
		SafeFlow	0.71	1.00	0.83
		SafeMFD	0.96	0.99	0.97
	t≤15 (84%)	EUnitigs	0.54	1.00	0.69
		SafeEPC	0.54	1.00	0.70
		SafeFlow	0.70	1.00	0.82
		SafeMFD	0.93	0.98	0.95
	All (100%)	EUnitigs	0.52	1.00	0.68
		SafeEPC	0.53	1.00	0.69
		SafeFlow	0.69	1.00	0.81
		SafeMFD	0.89	0.91	0.89

In the smaller *StringTie* dataset, *EUnitigs*, *SafeFlow*, and *SafeEPC* ran in less than 3 s, while *SafeMFD* took less than 20 min. Indeed, the ILP-based approach only ran out of time in four instances, which is also roughly 0.2% of the entire dataset.

In this dataset (see Table 1), the coverage is longer for all approaches. In particular, *EUnitigs* and *SafeEPC* reach a coverage of 68% and *SafeFlow* a coverage of 83%, while *SafeMFD* covers 92% of the transcripts in average. These improvements in coverage make all algorithms to obtain an *F*-score of over 80% and over 90% in the cases of *SafeFlow* and *SafeMFD*. Supplementary Fig. S3 breaks down these metrics by the number of ground truth paths.

In the harder RefSim dataset, *EUnitigs* and *SafeFlow* also ran in less than a second, while *SafeEPC* took approximately 2 min. In this case, *SafeMFD* ran out of time in 1562 graphs (15% of the entire dataset); however, recall that in these experiments we allow a time budget of only 2 min. In the rest of the dataset, it took approximately 7.5 h in total, corresponding to only 3 s on average per graph, again underlying that our method, even though it solves many NP-hard problems “per each input graph,” overall scales sufficiently well.

Table 1 shows that again *SafeMFD* dominates in coverage, being the only approach obtaining coverage over 89%, which is a 28% improvement over *SafeFlow*. This time its precision drops to close to 91%, and obtaining an *F*-score of 89%, which is a 9% improvement over *SafeFlow*. However, recall that coverage is computed only from “correctly aligned paths,” thus the drop in precision comes only from safe paths not counting in the coverage metric. If we restrict the metrics to graphs with at most 15 ground truth paths, which is still a significant proportion (84%) of the entire dataset, then *SafeMFD* has a very high precision (98%) while improving coverage by 32% with respect to *SafeFlow*. Thus, the drop in precision occurs in graphs with a large number of ground truth paths, which can also be corroborated in Supplementary Fig. S4.

These drops in precision (in all datasets) for large *t* can be explained by the fact that a larger number of ground truth paths produces more complex splice graphs and introduces more artificial solutions of potentially smaller size. As such, the larger *t*, the less likely that the ground truth is a MFD of the graph, and thus the more likely that *SafeMFD* reports incorrect solutions. This motivates future work on safety not only on MFDs but also in FDs of at most a certain size, analogously to how it is done for *SafeEPC*. This is still easily achievable with our framework by just changing the ILP blackbox, and keeping everything else unchanged (e.g. the inner and outer algorithms). Namely, instead of formulating the ILP model M(V,C) to admit solutions of exactly optimal *k* paths, it can be changed to allow solutions of “at most” $k^{\prime }$ paths, with $k^{\prime }\geq k$. If $k^{\prime }$ is also greater than the number of ground truth paths in these complex graphs, then safe paths are fully correct, meaning that we overall increase precision.

Table 2 shows the metrics’ improvements of adding subpath constraints. Provided with this additional information, *SafeMFD* significantly increases (3% on average) its coverage since longer regions are known to appear as part of the solution. On the other hand, precision also slightly increases (1% on average) as some incorrect solutions are discarded from the solution space. However, there is still a significant drop in precision for larger *t*.

**Table 2.** Summary of quality metrics in terms of nodes/exons for *SafeMFD* with subpath constraints.

Dataset	Max. Coverage	Wt. Precision	*F*-score
*Catfish*	0.88 → 0.89	0.99 → 0.99	0.93 → 0.94
*StringTie*	0.92 → 0.95	0.98 → 0.99	0.94 → 0.96
*RefSim*	0.89 → 0.92	0.91 → 0.92	0.89 → 0.91

*Note*: Each entry is shown in the format x→y, where *x* and *y* are the values for *SafeMFD* without and with subpath constraints, respectively.

## 5 Conclusion

RNA assembly is a difficult problem in practice, with even the top tools reporting low precision values. While there are still many issues that can introduce uncertainty in practice, we can now provide a major source of additional information during the process: which RNA fragments must be included in “any” parsimonious explanation of the data? Though others have considered RNA assembly in the safety framework (Khan *et al.* 2022a, Zheng *et al.* 2022), we are the first to show that safety can be practically used even when we look for optimal (i.e. minimum) size solutions. Our experimental results show that safe paths for MFD clearly outperform other safe approaches for the *Catfish* and *StringTie* datasets, commonly used in this field. On a significant proportion of the harder *RefSim* dataset, safe paths for MFD still significantly outperform other safe methods.

More generally, this is the first work to show that the safety framework can be practically applied to NP-hard problems, where the inner algorithm is an efficient test of safety of a group of paths, and the outer algorithm guides the applications of this test. Because our method was very successful on our test data, there is strong motivation to try the approach on other NP-hard graph problems whose solutions are sets of paths. For example, we could study other variations on MFD, such as finding FDs minimizing the longest path [NP-hard when flow values are integer (Baier 2004, Pieńkosz and Kołtyś 2015)]. The approach given in this article can also be directly extended to find decompositions into both cycles and paths (Dias *et al.* 2022a), though not trails and walks, because they repeat edges. We could also formulate a safety test for classic NP-hard graph problems like Hamiltonian path.

## Supplementary Material

btad640_Supplementary_Data
