# Supplementary material for: A safety framework for flow decomposition problems via integer linear programming
Source: Bioinformatics. 2023 Oct 20;39(11):btad640. doi: 10.1093/bioinformatics/btad640 (PMC10628435; doi:10.1093/bioinformatics/btad640)
Supplement: btad640_Supplementary_Data [file btad640_supplementary_data.pdf]

Supplementary Material:
A Safety Framework for Flow Decomposition Problems via Integer Linear Programming

Fernando H. C. Dias[1,⋆][0000−0002−6398−919X], Manuel Cáceres[1,⋆][0000−0003−0235−6951], Lucia Williams[2,⋆][0000−0003−3785−0247], Brendan Mumey[2,⋆⋆][0000−0001−7151−2124], and Alexandru I. Tomescu[1,⋆⋆][0000−0002−5747−8350]

[1] Department of Computer Science, University of Helsinki, Finland
{fernando.cunhadias,manuel.caceres,alexandru.tomescu}@helsinki.fi
[2] School of Computing, Montana State University, Bozeman, MT, USA
{luciawilliams,brendan.mumey}@montana.edu

A Additional Figures

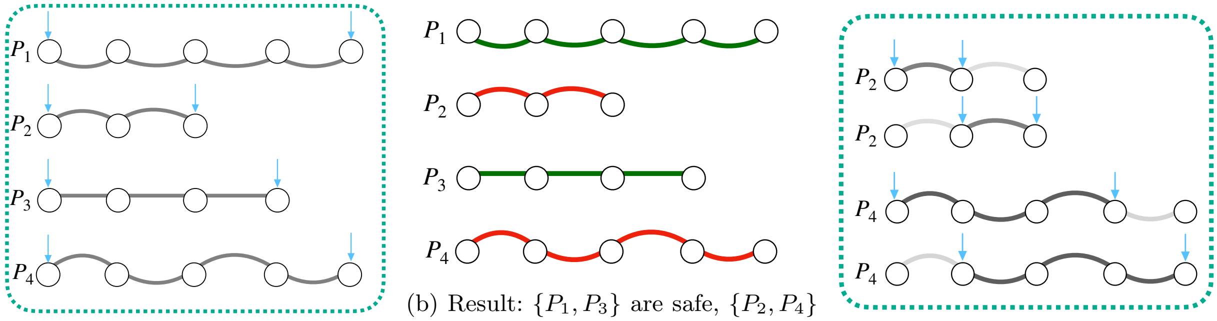

(a) First group test

(b) Result: $\{P_1, P_3\}$ are safe, $\{P_2, P_4\}$ are unsafe

(c) Second group test

Fig. 1: Illustration of the initial group tests performed by Algorithm 2. Fig. 1(a) shows the first group test (using Algorithm 1) on MFD solution paths $\{P_1, P_2, P_3, P_4\}$; suppose $\{P_1, P_3\}$ were safe (Fig. 1(b)); these are then reported as maximal safe. In this case we trim $\{P_2, P_4\}$ on both the left and right and make the next group test shown in Fig. 1(c).

B Additional Algorithms and Experimental Results

B.1 The bottom up algorithm

Algorithm 1, detailed below, uses a bottom-up group-testing strategy to find all maximal safe paths.

Definition 1. *We say a set of subpaths* $\mathcal{E} = \{P_i[l_i, r_i]\}$ *is an* extending core *provided all paths in* $\mathcal{E}$ *are safe and for any unreported maximal safe path* $P = P_i[l, r]$, *there is a* $P_i[l_i, r_i] \in \mathcal{E}$, *where* $l \le l_i \le r_i \le r$.

⋆ Shared first-author contribution
⋆⋆ Shared last-author contribution

Table 1: Summary of quality metrics in terms of genomic positions; t is the number of ground truth paths.

Dataset	Graphs	Algorithm	Max. Coverage	Wt. Precision	F-Score
StringTie	All (100%)	EUnitigs	0.78	1.00	0.87
		SafeEPC	0.79	1.00	0.87
		SafeFlow	0.88	1.00	0.93
		SafeMFD	0.95	0.98	0.96
RefSim	$t \leq 10$ (68%)	EUnitigs	0.72	1.00	0.83
		SafeEPC	0.73	1.00	0.84
		SafeFlow	0.84	1.00	0.91
		SafeMFD	0.97	0.99	0.98
	$t \leq 15$ (84%)	EUnitigs	0.70	1.00	0.82
		SafeEPC	0.71	1.00	0.83
		SafeFlow	0.83	1.00	0.90
		SafeMFD	0.96	0.98	0.97
	All (100%)	EUnitigs	0.68	1.00	0.80
		SafeEPC	0.69	0.99	0.81
		SafeFlow	0.81	1.00	0.89
		SafeMFD	0.93	0.91	0.90

Table 2: Summary of quality metrics in terms of genomic positions for *SafeMFD* with subpath constraints. Each entry is shown in the format $x \to y$, where x and y are the values for *SafeMFD* without and with subpath constraints, respectively.

Dataset	Max. Coverage	Wt. Precision	F-Score
StringTie	$0.95 \to 0.96$	$0.98 \to 0.99$	$0.96 \to 0.97$
RefSim	$0.93 \to 0.95$	$0.91 \to 0.92$	$0.90 \to 0.92$

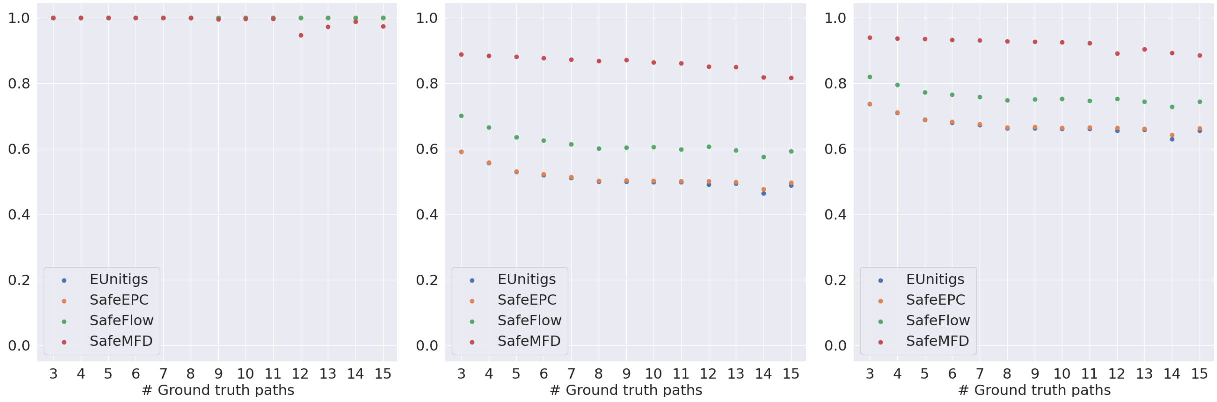

(a) Weighted Precision (b) Maximum Coverage (c) F-Score

Fig. 2: Quality metrics on graphs distributed by number of paths in the ground truth for the Catfish dataset. The metrics are computed in terms of exons/nodes.

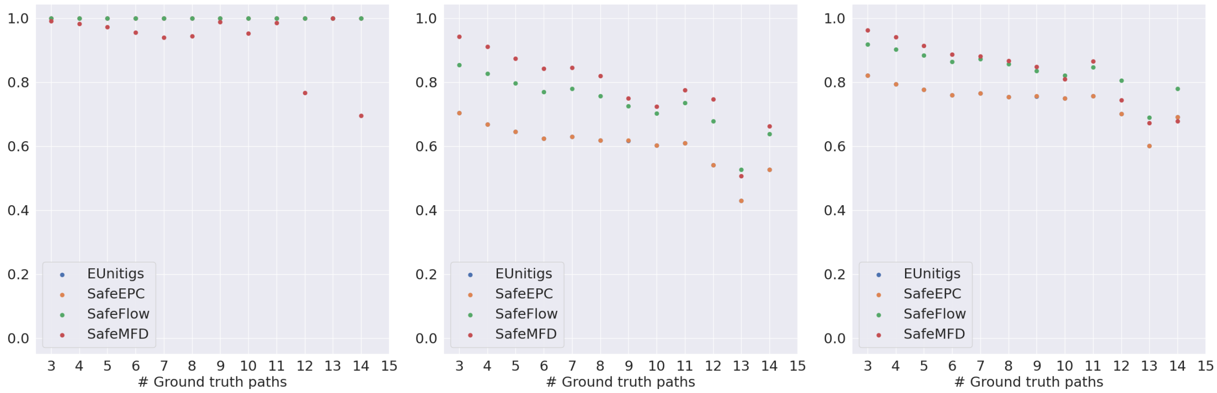

(a) Weighted Precision (b) Maximum Coverage (c) F-Score

Fig. 3: Quality metrics on graphs distributed by number of paths in the ground truth for the StringTie dataset. The metrics are computed in terms of exons/nodes.

Note that maximal FD-safe subpaths provide an extending core (as well just the set of all single-edge subpaths in each path). Algorithm 1 provides an algorithm to find all maximal safe paths based on group testing, starting from an extending core. The idea is to try both left-extending (by one) and right-extending (by one) each subpath in the core; if neither of these extensions are safe, then we know that that core subpath must be maximal safe. Testing all extensions can done quickly using Algorithm 1. We then recurse on a new core set consisting of those extensions that were found to be safe.

B.2 The two pointer algorithm

As we observed in Section 2.2.1, we can test whether a single path P is safe using one ILP call. We will assume that this test is encapsulated as a procedure $\mathsf{IsSafe}(\mathcal{M}, P)$. Once we can test whether a single path is safe for $\mathcal{M}(\mathcal{V},\mathcal{C})$, we can adopt a standard approach to compute all maximal safe paths. Namely, we start by computing one solution of $\mathcal{M}(\mathcal{V},\mathcal{C})$, $P_1,\ldots,P_k$ and then compute maximal safe paths by a two-pointer technique that for each path P_i, finds all maximal safe paths by just a *linear* number of calls to the procedure IsSafe [2].

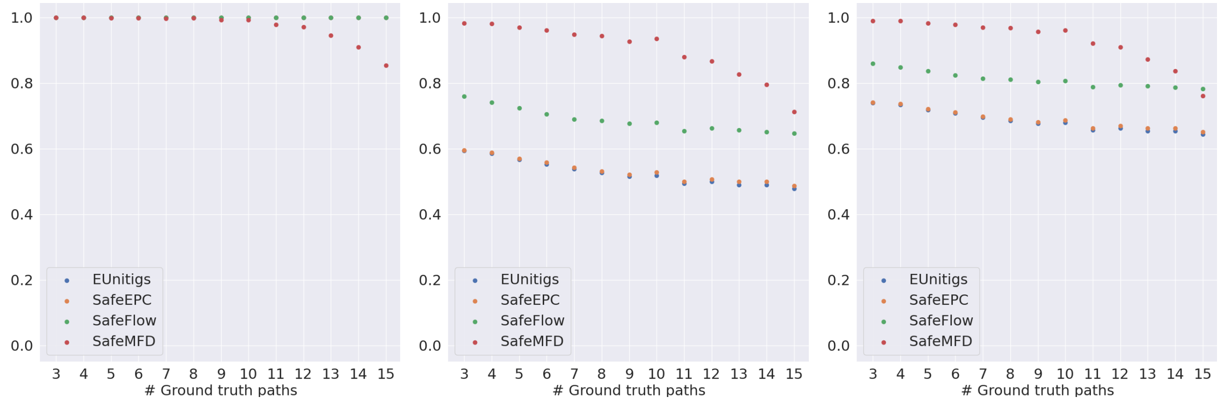

(a) Weighted Precision (b) Maximum Coverage (c) F-Score

Fig. 4: Quality metrics on graphs distributed by number of paths in the ground truth for the RefSim dataset. The metrics are computed in terms of exons/nodes.

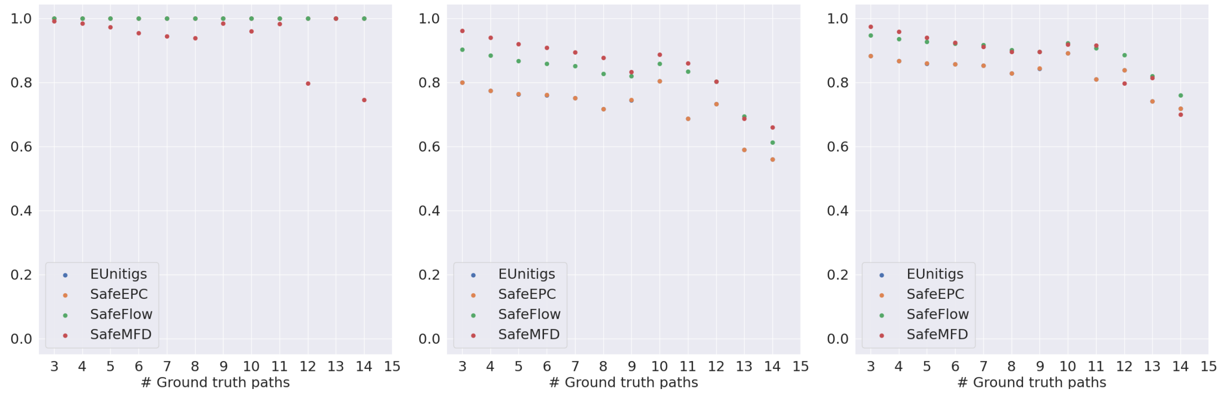

(a) Weighted Precision (b) Maximum Coverage (c) F-Score

Fig. 5: Quality metrics on graphs distributed by number of paths in the ground truth for the StringTie dataset. The metrics are computed in terms of genomic positions.

This works as follows. We use two pointers, a *left* pointer L, and a *right* pointer R. Initially, L points to the first node of path P_i and R to the second node. As long as the subpath of P_i between L and R is safe, we move the right pointer to the next node on P_i. When this subpath is not safe, we output the subpath between L and the previous location of R as a maximal safe path, and we start moving the left pointer to the next node on P_i, until the subpath between L and R is safe. We stop the procedure once we reach the end of P_i. We summarize this procedure as Algorithm 2; see also Figure 7 for an example.

B.3 Running time experiments among different variants proposed

We conducted the experiments on an isolated Linux server with AMD Ryzen Threadripper PRO 3975WX CPU with 32 cores (64 virtual) and 504GB of RAM. Time and peak memory usage of each program were measured with the GNU `time` command. *SafeMFD* was allowed to run `Gurobi` with 12 threads. All C++ implementations were compiled with optimization level 3 (*-O3* flag). Running time and peak memory is computed and reported per dataset.

SafeMFD includes the following four variants computing maximal safe paths:

TopDown : Implements Algorithm 2 using the group testing in Algorithm 1.

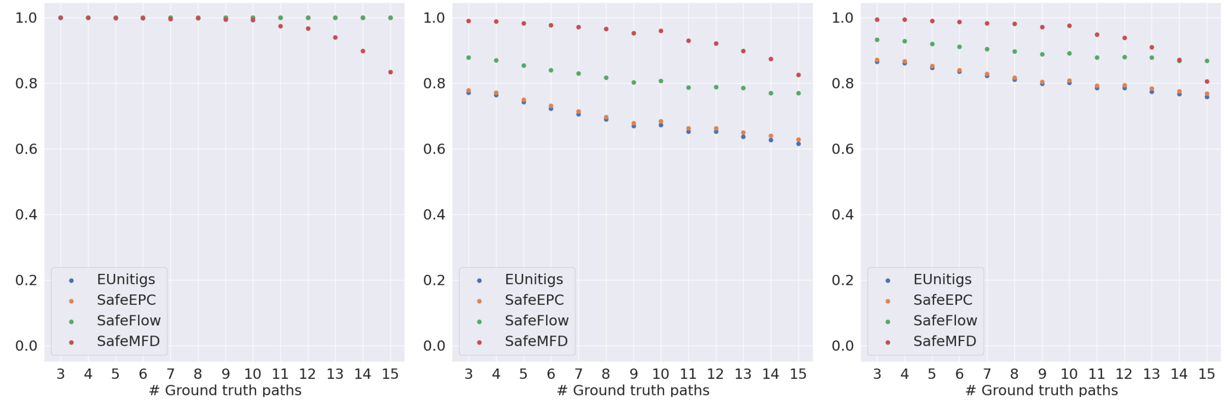

(a) Weighted Precision (b) Maximum Coverage (c) F-Score

Fig. 6: Quality metrics on graphs distributed by number of paths in the ground truth for the RefSim dataset. The metrics are computed in terms of genomic positions.

Algorithm 1: An algorithm to output all maximal safe subpaths that can be extended from an extending core set $\mathcal{E}$.

Input: An ILP model $\mathcal{M}$ and an extending core set $\mathcal{E}$

Output: All maximal safe paths for $\mathcal{M}$ that extend some path from $\mathcal{E}$

Procedure *AllMaxSafe-BottomUp*$(\mathcal{M}, \mathcal{E})$

  $\mathcal{L} = \{P_i[l_i - 1, r_i] : P_i[l_i, r_i] \in \mathcal{E}, l_i > 1\}$;

  $\mathcal{R} = \{P_i[l_i, r_i + 1] : P_i[l_i, r_i] \in \mathcal{E}, r_i < |P_i|\}$;

  $\mathcal{P} = \mathcal{L} \cup \mathcal{R}$;

  $\mathcal{S}$ = GetSafe$(\mathcal{M}, \mathcal{P})$;

  for $P_i[l_i, r_i] \in \mathcal{E}$ **do**

    if $P_i[l_i - 1, r_i] \notin \mathcal{S}$ **and** $P_i[l_i, r_i + 1] \notin \mathcal{S}$ **then**

      output $P_i[l_i, r_i]$;

  if $\mathcal{S} \neq \emptyset$ **then**

    AllMaxSafe-BottomUp$(\mathcal{M}, \mathcal{S})$;

BottomUp : Implements Algorithm 1 (Appendix B.1) using the group testing in Algorithm 1.
TwoPointer : Implements Algorithm 2 (Appendix B.2), the traditional two-pointer algorithm [2].
TwoPointerBin : Same as previous variant, but it additionally replaces the *linear scan* employed to extend and reduce the currently processed safe path by a *binary search*[3].

To compare between our four different variants we first run them all on our *harder* datasets: *Catfish* and *RefSim*, and then filter out those graphs that ran out of time in some variant. This way we ensure that no variant consumes its time budget and thus our running time measurements are not skewed by the unsuccessful input's timeouts. Applying this filter we removed 83 graphs from the *Catfish* dataset (0.3%) and 4,515 graphs from the *RefSim* dataset (43.74%).

Table 3 shows the running times and number of ILP calls of the different variants on both datasets. *TopDown* clearly outperforms the rest, being at least twice as fast, and performing (roughly) half many ILP calls. While *BottomUp* is analogous to *TopDown*, the superiority of the latter can be explained by the length maximal safe paths, indeed, since maximal safe paths are long it is faster to obtain them by reducing unsafe paths (*TopDown*) than by extending safe paths (*BottomUp* and both *TwoPointer* variants). On the other

[3] The binary search is only applied if the search space is larger than a constant threshold set experimentally.

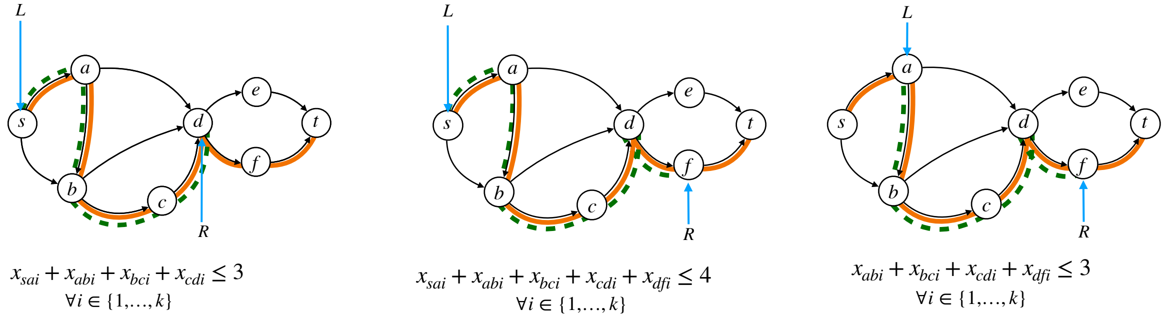

(a) Current iteration (b) Right pointer movement (c) Left pointer movement

Fig. 7: Illustration of the two-pointer algorithm applied on a flow decomposition path P_i (in orange). In each sub-figure, the subpath P (dashed green) between the nodes pointed by the left pointer L and the right pointer R is tested for safety, by adding constraints $\mathcal{S}(P)$. In (a), $\mathsf{IsSafe}(\mathcal{M}, P)$ returns *True*, and the right pointer advances on P_i. In (b), $\mathsf{IsSafe}(\mathcal{M}, P)$ returns *False*, and the previous subpath from (a) is output as a maximal safe path. In (c), the left pointer has advanced, and the new path P is tested for safety.

Algorithm 2: The two-pointer algorithm applied to compute all maximal subpaths of a given solution path P_i

Input: An ILP model $\mathcal{M}$ and one of its k solution paths, $P_i = (v_1, \ldots, v_t)$, $t \geq 2$
Output: All maximal safe subpaths of P_i for $\mathcal{M}$

```
Procedure AllMaxSafe-TwoPointer(M, P_i)
    L ← 1, R ← 2;
    while True do
        while IsSafe(M, P_i[L, R]) and R ≤ t do
            R ← R + 1;
        output P_i[L, R − 1];
        if R > t then return;
        while not IsSafe(M, P_i[L, R]) do
            L ← L + 1;
```

hand, *TwoPointer* is the slowest variant and, *BottomUp* and *TwoPointerBin* obtain similar improvements (over *TwoPointer*) by following different strategies. While *BottomUp* reduces the number of ILP calls more than *TwoPointerBin* (better appreciated in the RefSim dataset), the ILP calls of *BottomUp* take longer (since *BottomUp* tests several paths at the same time and *TwoPointerBin* only one), and thus the total running times of both is similar. This motivates future work on combining both approaches, while processing the paths starting from unsafe (as in *TopDown*) for better performance.

It could also be interesting to apply additional parallelization beyond that provided by allocation multiple threads to the Gurobi solver. There are two sources of parallelism that we we see: (1) in the group testing approaches, and (2) in the two-pointed algorithms. For (1), instead of testing a group of n paths, one could think of testing t groups of n/t paths, where t is the number of threads. However, note that while in the first case we have only one ILP call (with constraints for all t paths), in the second case we have t ILP calls, with constraints only for n/t paths). From our development experience, the running time of the ILP with constraints for less paths is of the same order as the one with all paths (since all contain at the core the ILP for the NP-hard MFD problem). As seen in Table 3, the key seems to be in reducing the number of ILP

Dataset (# Graphs)	Variant	Time (hh:mm:ss)	# ILP calls
Catfish (27,613)	TopDown	01:13:27	124,676
	BottomUp	03:22:13	212,774
	TwoPointer	04:21:44	226,365
	TwoPointerBin	03:31:57	216,540
RefSim (5,808)	TopDown	04:38:41	55,450
	BottomUp	11:55:20	76,837
	TwoPointer	13:48:00	127,352
	TwoPointerBin	11:34:02	119,218

Table 3: Running times and number of ILP calls in four different variants of *SafeMFD*.

calls. For (2), the two-pointer algorithms (which do not use group testing), can be naively parallelized by running them in parallel in each path of the initial MFD. However, note that in Table 3, after keeping only the graphs on which all tested methods complete, the best two-pointer algorithm is still three times slower than the fastest one (TopDown).

C Hardness of testing MFD safety

In this section we give a Turing-reduction from the UNIQUE 3SAT problem (U3SAT) to the problem of determining if a given path P in a flow network G is safe for minimum flow decomposition (call this problem *MFD-SAFETY*). A 3SAT instance belongs to U3SAT if and only if it has exactly one satisfying assignment. U3SAT has been shown to be NP-hard under randomized reductions [3], but it is open as to whether it is NP-hard in general.

The reduction leverages the construction in [1] that reduces 3SAT to minimum flow decomposition. We first briefly review this construction: A variable gadget (see Fig. 4 in [1]) is created for each 3SAT variable x and a clause gadget (see Fig. 5 in [1]) is created for each 3SAT clause. Positive literals in each clause receive flow from the left side of the corresponding variable gadget, whereas negative literals receive flow from the right side. Theorem VI.1 in [1] establishes that a 3SAT instance is satisfiable if and only if the constructed flow network has a minimum flow decomposition of a certain size. Any flow decomposition achieving this size must have a specific structure; in particular, there must be a flow path of weight 4 that either travels up the left side of the gadget (setting x to TRUE), or the right side (setting x to FALSE).

Theorem 1. *There is a polynomial time Turing-reduction from U3SAT to MFD-SAFETY.*

Proof. To obtain the desired Turing-reduction algorithm, instead of checking the size of the MFD, we will instead sequentially check the MFD-SAFETY of the aforementioned *side paths* traveling up the left and right sides of each variable gadget. Provided each variable gadget has exactly one safe side path we can then check the corresponding truth assignment to see if each clause is satisfied. If yes, we accept the instance as belonging to U3SAT, otherwise we reject.

Suppose the instance does belong to U3SAT. In this case there is a satisfying assignment so the MFD must have the structure as described above. Furthermore, since there is exactly one satisfying assignment, exactly one side path of each variable gadget must be safe and so our algorithm finds it and then verifies that the truth assignment satisfies each clause, thus accepting the instance. On the other hand, if the instance does not belong to U3SAT it could either be unsatisfiable or have multiple satisfying assignments. If unsatisfiable, no matter whether the safety checks pass, the corresponding assignment will not satisfy all clauses, so the instance will be rejected. If there are multiple solutions, then any variable that can be both TRUE and FALSE will not have a safe side path in the MFD. This means the safety check will fail and the instance will again be rejected. □

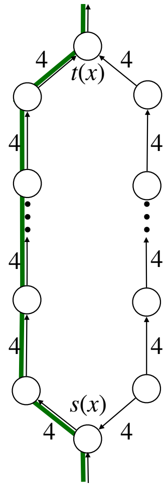

Fig. 8: The variable gadget from [1], showing only the weight 4 edges (other edges have weights from $\{1, 2\}$). A key property established in [1] is that if the 3SAT instance is satisfiable then in a minimum flow decomposition, a weight 4 flow path must travel up either the left side of the gadget (as shown), or the right side. A left flow path indicates the variable should be set to TRUE, while right indicates FALSE. We leverage this construction to reduce U3SAT to MFD-SAFETY.

References

1. Tzvika Hartman, Avinatan Hassidim, Haim Kaplan, Danny Raz, and Michal Segalov. How to split a flow? In *2012 Proceedings IEEE INFOCOM*, pp. 828–836. IEEE, 2012.
2. Shahbaz Khan, Milla Kortelainen, Manuel Cáceres, Lucia Williams, and Alexandru I Tomescu. Improving RNA assembly via safety and completeness in flow decompositions. *Journal of Computational Biology*, 2022.
3. L.G. Valiant and V.V. Vazirani. NP is as easy as detecting unique solutions. *Theoretical Computer Science*, 47:85–93, 1986.
